# Robert K. Smither

**Published:** 1917-07

**Authors:** 


**Robert K. Smither**, a prominent pharmacist of Buffalo, died at his home May 24, after an illness of five days. He was born in Winchester, Eng., Oct. 10, 1851, came to Canada with his parents when 7 years old and had his first experience in pharmacy in Brampton, Ont. He came to Buffalo as a young man and joined the staff of W. H. Peabody at the corner of Main and South Division Streets. Later, he became manager of the W. R. Crumb pharmacy at the corner of Niagara and Carolina Streets, and, in 1875, opened a store of his own at the corner of Niagara and Jersey Streets. Still later, he formed a partnership with George I. Thurstone and established the Smither-Thurstone Pharmacy at the corner of Elmwood Avenue and Bryant Street, and, independently, the Parkside Pharmacy at Main Street and Leroy Avenue. The last three stores are still in operation. He served as supervisor from the old 9th ward for three terms from 1879 and as alderman from the 24th ward, for six years from 1891, being president of the board in 1895.

